# A Case Study of Community Involvement Influence on Policy Decisions: Victories of a Community-Based Participatory Research Partnership

**DOI:** 10.3390/ijerph13050515

**Published:** 2016-05-20

**Authors:** Edith M. Williams, Julien Terrell, Judith Anderson, Laurene Tumiel-Berhalter

**Affiliations:** 1 Department of Public Health Sciences, Medical University of South Carolina (MUSC), 135 Cannon Street, Charleston, SC 29425, USA; 2 The Brotherhood/Sister Sol (Bro/Sis), 512 West 143rd Street, New York, NY 10031, USA; julien@brotherhood-sistersol.org; 3 Environmental Justice Action Group (EJAG) of Western NY, Buffalo, NY 14203, USA; judie851@aol.com; 4 Primary Care Research Institute, University at Buffalo, UB Gateway Building, 77 Goodell St, Suite 220, Buffalo, NY 14203, USA; tumiel@buffalo.edu

**Keywords:** Lupus, Superfund, community-based participatory research, health policy

## Abstract

The Buffalo Lupus Project was a community-university partnership that investigated associations between exposure to a local waste site and high rates of lupus and other autoimmune diseases. The partnership’s major accomplishment was successful advocacy for containment and clean-up of the site. As a result of community education, the remediation plan suggested by the community was adopted. Additionally, when a local childhood lead poisoning testing program was canceled, community members signed a letter to legislators urging them to replace the funding, which was restored within one week. This demonstrated how coordinated community-based capacity-building efforts can influence health policy.

## 1. Introduction

### Community-Based Participatory Research: A Historical Perspective

Many racial/ethnic and low socio-economic status (SES) populations are disproportionately burdened by poverty, environmental injustice and health disparities [1,2,3,4,5,6,7,8,9,10,11,12,13,14]. Community-based organizations (CBOs) in communities of color struggle to trust or participate in government and university research that they believe underestimates and under-values deaths, suffering, and solutions in their communities. As a result, some organizations have pushed academic institutions to make the research process more participatory, equitable, and action-oriented [15,16,17,18,19,20]. Environmental justice (EJ) organizations have engaged in community-based participatory research (CBPR) as a way to address local EJ and health issues [16,21,22,23,24,25] rather than relying on the efforts of traditional research to provide evidence that can be translated into effective policies and solutions.

Communities across the nation have employed CBPR in partnerships with universities and other stakeholders and demonstrated their capacity to perform environmental surveillance to measure their exposure to pollution and unhealthy land uses and the resulting health effects. In New York, community groups have performed environmental surveillance to address asthma and risks from subsistence-fish diets. The West Harlem Environmental Action (WE-ACT) has collaborated with researchers at Columbia University to measure and map air pollution from factories and vehicle exhaust and to address asthma as well [26,27,28,29]. The Bucket Brigade in Louisiana, a leader in community-driven surveillance of air pollution, has used low-cost sampling methods to measure toxic air pollution levels near overburdened and poor neighborhoods and African American communities in Louisiana’s Cancer Alley, a corridor of petrochemical companies along the Mississippi River from Baton Rouge to New Orleans [30,31]. Similar work is being performed by groups in Oakland, California, to assess human exposure to diesel exhaust and other contaminants using cumulative risk assessment methodology [32]. In North Carolina, the West End Revitalization Association has been a leading authority on the surveillance of built environment insults related to inequities in zoning, planning, and community development, as well as infrastructure disparities in underserved African American neighborhoods. Because of its efforts to collect data on the quality of sewer, water, and road infrastructure to obtain legal compliance, underserved residents have received paved roads and increased access to sewer and water services [33,34,35,36]. The Concerned Citizens of Tillery was one of the first community-based organizations (CBO)s to participate in surveillance research on the impact of industrial hog farms on neighborhood air quality and human health [25,37]. The National Institute of Environmental Health Sciences, the Environmental Protection Agency, and the National Institute of Occupational Health and Safety played an instrumental role in funding environmental justice programs that brought together scientists and communities as demonstrated by a supplement to the *American Journal of Public Health* published in 2009 [38].

This case study describes a multi-faceted community-based participatory research program and the tangible impacts the community-university partnership had on health policy. The Standards for Reviewing Qualitative Studies (SRQS) were used as a guideline for preparing this manuscript [39].

Members of disadvantaged communities of color in Buffalo, New York, organized to address their exposure to multiple sources of pollution in the Buffalo region, following the civic engagement model of other EJ and CBO efforts. Concerned community stakeholders who were part of the Citizen’s Environmental Coalition (CEC) developed the Toxic Waste/Lupus Coalition in 1993, which subsequently assisted in organizing the Coalition of Impacted Neighborhoods (COIN), a Western New York action group. This group formed to address the identified health concerns, which were primarily the alarmingly high rates of lupus and other autoimmune diseases in the area. Door-to-door canvassing revealed a cluster of 17 women with Systemic Lupus Erythematosus (SLE) living in one eight-block area in zip codes 14211 and 14215, which is a largely African American area of Buffalo, New York. With further investigation, the original cluster eventually grew to 68 African American women and men with a history of residence in zip codes 14211 and 14215. Three toxic waste sites were also identified within or in close proximity to the two zip codes. Two had been addressed through remediation or the reversal of environmental damage (commonly achieved by excavating a specified area of contaminated soil and replacing it with clean soil), but the largest had been designated as a level two Superfund site that had been uncontained for several years waiting for State Superfund Program funding. Community members were concerned that there were no fences or signs indicating hazardous waste to protect residents from being unknowingly exposed to contaminants in soil and water around the site. Additionally, the site was directly across the street from a church with a congregation of over 3000 members and an elementary school with 700 students. 

In 2000, the Toxic Waste/Lupus Coalition approached the University at Buffalo as well as the New York State Department of Environmental Conservation (NYS DEC) for expert advice on how to address their concerns. Based on results of the NYS DEC investigation in 1997–1998, they determined that contaminants present on-site included lead, incinerator ash, Volatile Organic Compounds (VOCs), and polychlorinated biphenyls (PCBs). The DEC conceded that PCB contamination was limited to an area of 750 square feet, caused by dumping or spillage by the lead smelting plant that had operated there in the past. Substantial lead contamination was identified at subsurface levels between 110 and 46,700 ppm and surface levels between 149 and 11,500 ppm. A Record of Decision (ROD) was issued in March 1999 which selected a remedy that called for the excavation and removal of the contaminated soil from the site to an approved landfill. 

The University at Buffalo entered into a memorandum of understanding (MOU) in December 2000 with the community coalition and developed a collaborative research proposal to study high rates of asthma and autoimmune diseases in minority communities of Buffalo, New York. The Buffalo Lupus Project was part of the larger CBPR grant that was awarded in 2001 and was funded by the National Institute of Environmental Health Sciences (NIEHS) in the amount of $1.3 million over five years.

**Figure 1:**
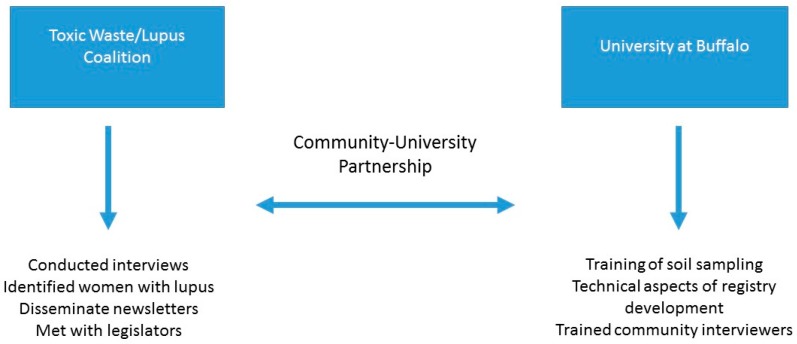
Details the roles and responsibilities of the Toxic Waste Coalition and the University at Buffalo.

The Buffalo Lupus Project expanded on findings of contamination by proposing to identify community residents with lupus and other autoimmune diseases and assess whether exposure to the local Superfund site was related to the high incidence of lupus and other autoimmune diseases in the area. The project took a multi-faceted approach to the impacts of pollution on the environment and the health of a maximally exposed population in the Buffalo area by employing the CBPR framework [33,34] to: (1) eliminate toxic waste sites in the African American community specifically and all other impacted communities; (2) educate the community on the toxic waste issue; (3) mobilize the community to get the sites cleaned up; (4) educate the community on how the toxic wastes may contribute to the increase of people being affected by autoimmune deficiency diseases; (5) develop a collaborative study with the University at Buffalo to assist in addressing concerns; (6) create relationships with other environmentally conscious groups throughout the state of New York and the nation; (7) develop and strengthen partnerships with all governmental agencies such as the Department of Environmental Conservation (DEC), Department of Health (DOH), and all governmental officials; and (8) bring community outreach to neighborhood block clubs, groups, community-based organizations, churches, and schools. Figure 1 details the roles and responsibilities undertaken by each of the partners to reach these goals.

## 2. Experimental Section 

### Research Efforts

To accomplish the Year 1 project aim of assessing the status of the waste sites and point sources of pollution, citizens banded together with other organizations in affected communities for a variety of education, training, and outreach activities [40]. Other Year 1 and 2 aims, including identifying and characterizing populations affected, evaluating the identified cluster of lupus, improving communication vehicles, and improving the city-wide surveillance system for autoimmune diseases, were also addressed. A registry was pursued to assess how many people in the community had lupus or another similar disease using an extensive media campaign as well as word-of-mouth within the community. Additionally, phone and in-person surveys were administered by trained interviewers with registry participants with SLE who had ever resided in the zip codes in closest proximity to the site of concern. The purpose was to uncover any common factors that could help to explain the complex causes of lupus and other similar diseases. Survey questions included residential, occupational, and medical histories. 

**Figure 2:**
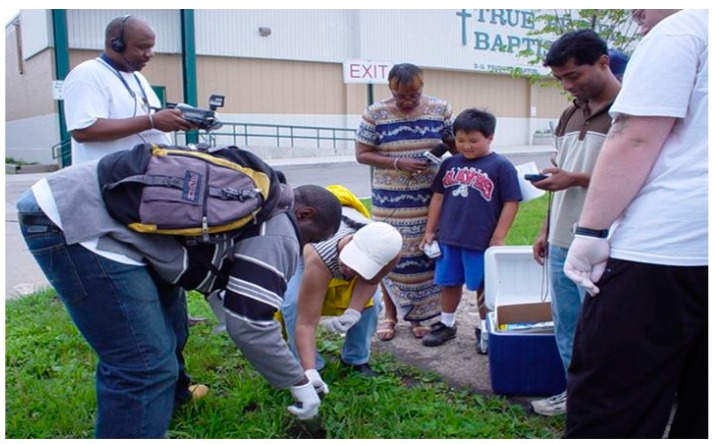
Community members taking soil samples for analysis.

Newsletters were disseminated throughout the community, informing them of research activities until the partnership began to hold regular community meetings. As a result of continued community efforts and regular meetings with local legislators, resolutions were passed to have the sight secured by fencing and warning signs, which were erected by the city in 2000, followed by a resolution to clean up the site in 2002. That same year, the chemistry department at the University at Buffalo became involved in research activities. Researchers were concerned that due to the dimensions of the site, there was a possibility that toxic chemicals could migrate off site by wind or children playing there during the years prior to fencing and signage. Based on those concerns, soil samples were taken in front of the site and across the street in front of the church. Samples were also taken up the street at a municipal housing project and on adjacent streets to assess the potential extent of toxin migration. As shown in Figure 2, community members were taught how to take the samples and were supervised by university chemists [41].

## 3. Results

### 3.1. Sampling Results

Sampling revealed that mercury and arsenic levels were not high enough for remediation concern, but suggested that the results should be discussed. Lead levels were very low on the municipal housing site, and lead levels were typical for adjacent streets and not of concern, although some simple clean-up was recommended. However, lead levels on the street side of the site were quite high, which confirmed that this area needed to be considered for remediation. Based on the results of the expanded investigation, the recognized volume of contaminated soil increased from 3575 cubic yards at the site to a total of 87,200 cubic yards for properties that were both on and off of the site (e.g., land adjacent to the site and land across the street where the church and school were located) to be stabilized there and subsequently moved to a landfill for hazardous waste.

### 3.2. Capacity-Building 

To improve the capacity of the community, the community-university partnership provided community members with the information and skills necessary to be involved in the process of site remediation. Additional stakeholders were recruited into the coalition and community members were educated about toxic waste, other forms of pollution and the cumulative impact that occurs from exposure to the many different sources in their neighborhood. Health impacts discussed focused on community-specific concerns such as extremely high levels of lupus and other similar diseases such as Multiple Sclerosis (MS), rheumatoid arthritis (RA), and type 1 diabetes, as well as asthma and lead poisoning.

Community-university partnership committees were comprised of community members and university faculty and students. All committees were chaired by community members, giving them the power to shape outreach efforts and research tools. In the committee structure, the University at Buffalo and the Community Coordinator fell below community members and other stakeholders. The University at Buffalo had its own framework, and the Community Coordinator was responsible for seeking additional funds and resources, networking and coalition building, and facilitating the overall agenda. The Communication and Publicity Committee supervised the communication of the agreed-upon message through media, the Information and Stakeholder Development Committee identified research money outside of the university, and the Survey Coordination and Training Committee coordinated survey administration and training of those collecting the information. Additionally, the Research and Survey Committee and staff were required to take the National Institutes of Health (NIH) online Human Participation in Research certification course. Educational workshops were held and fact sheets created on DEC policy and procedures. Articles were also published in the Coalition’s newsletter about the site, health impacts and Coalition activities. Community presentations and articles were also prepared and delivered on environmental justice and community issues and impacts.

On 29 June 2005, the DEC held a public meeting where five remediation plans were presented for public comment. Since most of the community was unfamiliar with the DEC and its policies and procedures, prior to the meeting the community held an educational session, published an article in the Coalition’s newsletter, and produced a public meeting fact sheet on the DEC’s public meeting process, empowering community members to come to the meeting prepared to ask relevant questions. The five remediation options offered by the DEC are presented in Table 1.

**Figure 3:**
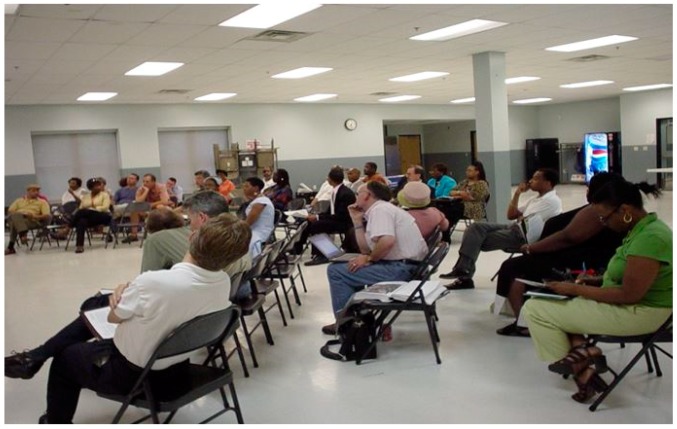
Community members attending a public meeting to discuss remediation plans.

As shown in Figure 3, 50 community members attended the public meeting, and due to their increased knowledge and expertise, they suggested the following sixth alternative remediation plan:

Remove contaminated soil from the site to achieve a clean-up goal used for unrestricted future use and remove contaminated soils from off-site properties to achieve a clean-up goal used for industrial/commercial use as it is currently zoned.

The Coalition had also gotten a letter from the City of Buffalo’s Office of Strategic Planning committing to turn the land over for whatever use the community wanted after the remediation. These actions were critical to the DEC’s decision-making process and the community’s recommendation was adopted and remediation activities were initiated.

### 3.3. Continued Community Activism

Besides serving as a catalyst for the clean-up of the site by the Department of Environmental Control (DEC) and Department of Health (DOH), the project helped address other environmental concerns that were not related to the original aims of the study. During remediation activities, a county budget crisis led to the elimination of funding for childhood lead screening. This enraged the community because three zip codes in Buffalo had the highest rates in New York State of lead incidence in children between 1996 and 1999. Those zip codes were 14208, 14209, 14212 and 14211, which is the location of the 858 East Ferry Street Superfund site [42,43].

Community members mobilized. They contacted the Erie County Health Department to find out specifically how this would impact the screening process and how many children this could potentially harm. They protested the budget cut in front of the Erie County Hall and held a locally televised press conference, which included expert testimony on issues related to environmental justice and impacts of lead poisoning. The church in close proximity to the site also took action. The church’s pastor asked his congregation to call their County Legislators and request that the funding for childhood lead screening be restored. As a result, more than 1000 calls were made to the county legislature. Soon after that, county legislators announced a resolution to restore childhood lead screening to the County Budget. Ten days later the funding was back in place.

## 4. Conclusions

People of color have suffered environmental injustices for several decades. Inhabitants of this largely African American neighborhood in Buffalo, New York, lived, worked, played, and prayed on top of 87,200 cubic yards of lead-, mercury-, and arsenic-laden soil. This community of color knew there was an unusual trend of illness in their community, felt there was an identifiable cause, and sought out the knowledge/expertise they knew were needed to uncover it. Through the process of community-based participatory research (CBPR) and the partnership with the University at Buffalo, a framework was mutually established to inform, educate and empower the community to take control of their own destiny.

Using this knowledge to develop news articles and educational workshops for community residents helped them understand environmental impacts on the health of community members and build the skills necessary to implement change. That knowledge resulted in the increased capacity of community members to perform research on issues of concern and present findings in appropriate contexts, along with the substantiating data to the designated agency. Department of Environmental Control (DEC) policy mandated that the clean-up be limited to the level commensurate with the zoning of the property unless there was an approved plan for redevelopment that required a higher level of clean-up. Through this process of community engagement, building relationships, and targeted capacity-building, DEC policy and procedures were amended. Additionally, because of the information received at various community meetings, workshops and Coalition newsletters, a member of the community who now knew where to go and how to take action picked up childhood lead screening in a long list of county budget cuts. 

Members of disadvantaged communities of color in Buffalo, New York, organized to address their exposure to multiple sources of pollution in the Buffalo region following the civic engagement model of other environmental justice (EJ) and community-based organization (CBO) efforts. Bringing the information to the community at large with the research and data supplied by experts in the affected fields was the catalyst to informing and mobilizing the community. The accomplishments of the Buffalo Lupus Project demonstrate that communities can affect policy decisions if they are organized, informed and committed to the issue and the process. Our findings have important implications for pollution prevention, risk reduction activities and strategies, and environmental health policy for other economically disadvantaged and overburdened communities. 

## Figures and Tables

**Table 1:** DEC Remediation Options.

Remediation Option	Cost	Action
1. No Action	$0	Remain hazardous with no use
2. Soil excavation and removal to a landfill with a cap on site	$35 million	industrial use
3. Partial excavation and consolidation	$1.6 million	Cap waste on site with some beneficial use
4. On-site soil washing technology	$50 million	Unrestricted use on and off site
5. Excavation of soils from off site, partial consolidation on site and partial disposal	$16 million	On site with cap for industrial use and some beneficial use
